# Integrated Neuromuscular Inhibition Technique Versus Mulligan Mobilization on Functional Disability in Subjects With Nonspecific Low Back Pain: A Comparative Study

**DOI:** 10.7759/cureus.30253

**Published:** 2022-10-13

**Authors:** Neha Chitale, Deepali S Patil, Pratik Phansopkar

**Affiliations:** 1 Department of Musculoskeletal Physiotherapy, Ravi Nair Physiotherapy College, Datta Meghe Institute of Medical Sciences, Wardha, IND; 2 Department of Physiotherapy, Ravi Nair Physiotherapy College, Datta Meghe Institute of Medical Sciences, Wardha, IND

**Keywords:** chronic nonspecific low back pain, nonspecific low back pain, integrated neuromuscular inhibition, mulligan mobilization, chronic low back pain (clbp)

## Abstract

Background

Pain lasting more than three months is termed chronic pain. Treating chronic pain is always a challenge for the therapist. Low back pain (LBP) with a high prevalence is a point of concern. Various treatment methods are available. The two treatment methods are integrated neuromuscular inhibition technique (INIT) and Mulligan mobilization with movement (MWM). In this study, we have compared INIT with MWM.

Method

It was an interventional study carried out at Ravi Nair Physiotherapy College and Acharya Vinoba Bhave Rural Hospital. A total of 80 participants with nonspecific LBP were included in the study. The participants were randomly divided into two groups and treated for two weeks with three weekly sessions.

Statistical analysis and result

Statistical analysis was done post the completion of sampling. Paired and unpaired t-tests were used. A p-value of <0.05 was considered significant. The result was obtained after comparing the pre- and post-values of the numerical pain rating scale (NPRS), modified Oswestry disability index (MODI), and range of motion (ROM) of the lumbar joint. After two weeks of treatment, a reduction in functional disability and pain was seen in the INIT and MWM groups. ROM was increased after two weeks of treatment in both INIT and MWM groups. When compared, INIT showed better results than MWM.

Conclusion

In conclusion, we saw that the integrated neuromuscular inhibition technique might be a better technique than Mulligan mobilization with movement in terms of reducing pain and functional disability.

## Introduction

The spine is constructed out of vertebrae, as well as intervertebral discs. The mobility is granted by the intervertebral discs, which will not endanger the vertebral column's supportive strength [1]. The human vertebral column consists of 33 vertebrae classified as cervical, thoracic, lumbar, sacral, and coccyx [2,3]. For postural control and spinal stability, the spine is connected to the trunk's muscles and ligaments [4,5]. The lumbar segment comprises five vertebrae named L1-L5 [6]. They have a thick spinous process and large vertebral bodies in comparison to the size of the vertebra. The spinous process protrudes perpendicular to the body. The facets are curved articular surfaces [1,7]. As the lumbar region is less mobile and has a vast muscular structure, any derangement leads to pain and hampers the activities of daily living [8].

With the prevalence of eight and two-tenths percent worldwide in 1990 to seven and five-tenths percent in 2017, low back pain (LBP) needs attention [9]. People suffering from LBP should manage pain earlier to avoid biomechanical alteration. The low back pain where the cause is not known is nonspecific low back pain (NSLBP), and when the cause of pain is specific, it is referred to as specific low back pain. The pain that is mechanical in origin is mainly because of the muscles or the intrinsic factors; it is termed mechanical low back pain [10]. Mechanical low back pain is experienced due to sudden heavy lifting or prolonged sitting. Nerve root-associated LBP is due to the nerves getting compressed at the spinal level; the pain is sharp and burning in nature, radiating to the nerve course. Pathologic LBP is due to underlying pathology, which can be a pathology of the bones, muscles, or intervertebral disc. The prolapsed intervertebral disc is a common cause of LBP [2].

The treatment of LBP depends upon the cause of pain. So, therapists should use various maneuvers and identify the best method to manage low back pain. Integrated neuromuscular inhibition technique (INIT) and Mulligan mobilization with movement (MWM) are two techniques that can be used to manage LBP [11].

INIT is a method that includes three maneuvers in one [12]. The three techniques are trigger point release [13], strain counterstrain technique [14], and muscle energy technique (MET) [15]. In trigger point release, compression is given at the trigger point region and maintained for 15 seconds, while in strain counterstrain technique, the superficial fascia is stretched. MET works on the principle of reciprocal inhibition. MWM is a type of mobilization done with movement, which helps reduce joint stiffness [16]. Mulligan is used for both peripheral and spinal joints. Sustained natural apophyseal glide (SNAG) is the type of movement in which the subject performs active movement while the therapist applies sustained pressure on the area of hypomobility. Natural apophyseal glide is when the glide is involved but there is no functional movement taking place [17].

INIT has been proven effective in treating neck pain, but no pieces of evidence are seen for the low back region yet. Mulligan mobilization with movement is proven effective in reducing pain in the lower back region. This study will be conducted to see if integrated neuromuscular inhibition effectively reduces functional disability and pain in nonspecific low back pain and which technique out of INIT and MWM is better.

## Materials and methods

This experimental trial was performed at Ravi Nair Physiotherapy College, Datta Meghe Institute of Medical Sciences (DMIMS), Sawangi (Meghe), Wardha, musculoskeletal outpatient department. Approval was from the Institutional Ethical Committee of Datta Meghe Institute of Medical Sciences (DMIMS, Deemed to be University {DU}), Sawangi (Meghe), Wardha (DMIMS (DU)/IEC/2021/241) and the Clinical Trials Registration of India (CTRI/2021/05/033461). Participants who gave consent to participate were included in the study. A total of 80 individuals with nonspecific chronic LBP were included using simple random sampling. Each group had 40 individuals, and allocation was done using envelop method.

Inclusion and exclusion criteria

Participants of both genders aged 18-25 with no neurological impairment and willingness to participate were included in the study. Participants treated for LBP with some form of surgical intervention or with a history of trauma to the back region or acute pain or individuals with lumbar radiculopathy were excluded.

Procedure

Participants were screened according to the inclusion and exclusion criteria. Those who fulfilled the requirements and were willing to participate were included in the study. They were divided into groups A and B. Group A received INIT, whereas group B received Mulligan SNAG. INIT and Mulligan SNAG followed standard protocol for treatment. Both groups received conventional physiotherapy protocol, which included interferential therapy for 20 minutes [18] and back strengthening exercises [19,4]. The treatment was provided by the physiotherapist for two weeks with three sessions per week. Modified Oswestry disability index (MODI) [20-22], numerical pain rating scale (NPRS) [23,24], and modified Schober's test [25,26] were taken as outcome measures before the treatment and at the end of the treatment.

## Results

Statistical analysis was performed using descriptive and inferential statistics, including Student's paired and unpaired t-test and the software Statistical Product and Service Solutions (SPSS) (IBM SPSS Statistics for Windows, Armonk, NY) 27.0 version and GraphPad Prism (GraphPad Software, San Diego, CA) 7.0 version, with a p-value of less than 0.05 assumed to be the significance level. Paired t-test was used to compare pre-data with post-data within the group, while an unpaired t-test was used to compare the post-mean between the groups in age-wise distribution. The mean value of the INIT group is 21.3, with a standard deviation of 2.5, while the mean value of the Mulligan group was 21.46, with a standard deviation of 2.202. According to gender, in two groups, out of 40 patients in the INIT group, 28 were male, and 12 were female. In the Mulligan group, 24 male participants were included and 16 females.

**Figure 1:**
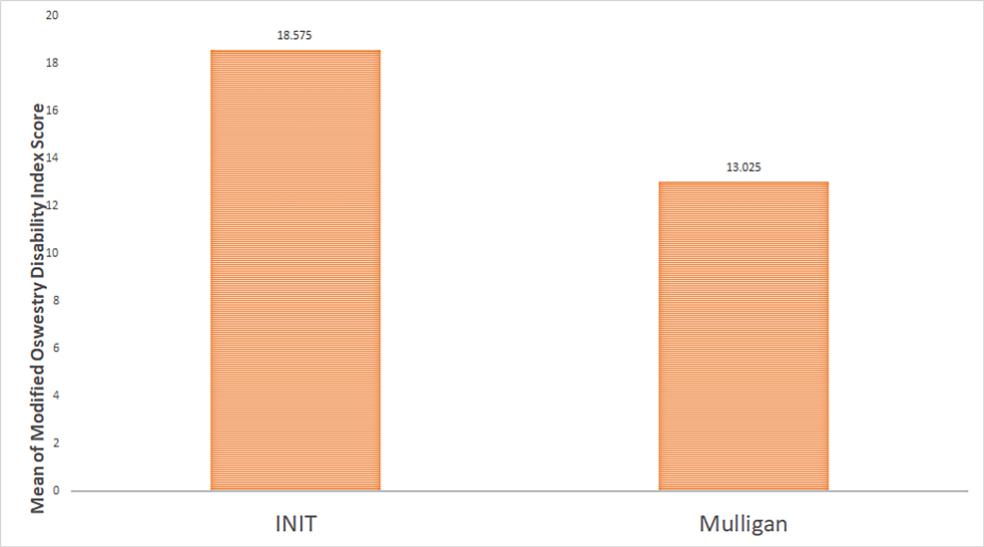
Comparison of mean difference in modified Oswestry disability score in two groups INIT: integrated neuromuscular inhibition technique

**Figure 2:**
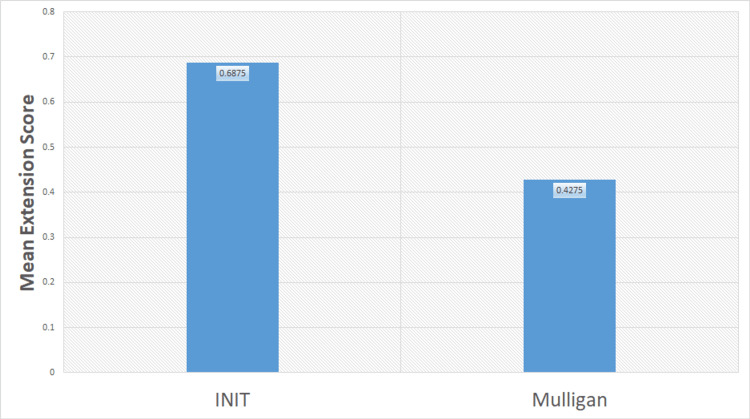
Comparison of mean difference in lumbar extension score in two groups INIT: integrated neuromuscular inhibition technique

Statistical analysis and the effect of treatment on both groups are shown in Table 1 and Figures 1-2. Mean, standard deviation, and standard error in group A and group B for NPRS, MODI, and range of motion (ROM) are mentioned in Table 1. The mean difference in the INIT group was significantly reduced in NPRS, MODI, and ROM. INIT outperformed MWM demonstrating a significant difference between the two treatments. The mean difference of MODI in the INIT and Mulligan groups was 18.575 and 13.025, respectively (Figure 1). The mean difference in lumbar extension in INIT and Mulligan groups was 0.6875 and 0.4275, respectively (Figure 2).

**Table 1:** Comparison of mean difference in NPRS, MODI, lumbar flexion, and lumbar extension in two groups NPRS: numerical pain rating scale; INIT: integrated neuromuscular inhibition technique; MODI: modified Oswestry disability index; N: number of samples

Group	Outcome measure	N	Mean	Standard deviation	Standard error mean	T-value	P-value
INIT	NPRS	40	2.125	0.812	0.13	2.3933	0.0191
Mulligan		40	1.675	0.8482	0.14		
INIT	MODI	40	18.575	5.8647	0.94	3.2102	0.003
Mulligan		40	13.025	9.065	1.45		
INIT	Lumbar flexion	40	0.9275	0.6364	0.102	4.3808	0.001
Mulligan		40	0.415	0.3589	0.057		
INIT	Lumbar extension	40	0.6875	0.4697	0.075	2.9065	0.0047
Mulligan		40	0.4275	0.3025	0.048		

## Discussion

The study was conducted to explore the effect of INIT and Mulligan lumbar SNAGs on pain and disability in NSLBP and to compare the effect of INIT and Mulligan lumbar SNAG. A population aged 18-25 was selected to avoid degenerative changes, which occur with advancement in age. The exclusion included any deformities, tumors, new or old fractures, pregnancy, surgeries around the back and thorax, and radiculopathies or neurological defects. Participants were equally divided into two groups, 40 per group. The therapist gave interferential therapy and back-strengthening exercises to both groups. Group A received INIT, while group B received Mulligan lumbar SNAG. The treatment was given for two weeks with three sessions in one week, so the therapist gave a total of six sessions to each patient. Pain, disability, and the range of motion were monitored using NPRS, MODI, and modified Schober's test, respectively.

LBP was considered for this study as it also appears to be frequent in youngsters [26]. Nonoccupational intensive physical exercise has been linked with an enhanced prevalence of LBP, although recreational activities were already linked to a protective effect. It is a symptom rather than a disease: the pain is the product of several pathologic practices that result in this recurring condition. The human spine is a complex structure that holds us upright and allows us to move in various directions in space; it is also subject to numerous forces working on our body, whether we stand, walk, lift, carry, or push/pull weights [27]. Several components in the system might produce pain. However, without invasive validation, it may be challenging to attribute pain to a specific structure (or structures) in a given case [28,29]. Some jobs need extended periods spent in one posture. This leads to the weakening and tightening of some muscles, leading to lower cross syndrome resulting in back pain. This prolonged posture leads to musculoskeletal disorders forming trigger points in specific muscles. Trigger points may manifest a decreased range of motion and LBP [30].

Tawrej et al. studied the effect of MET on quadratus lumborum muscle in subjects with NSLBP. The therapist gave a hot pack along with MET. The author concluded that thermotherapy increases soft tissue flexibility. The author added that hot fermentation could reduce LBP by blocking the pain signal [31]. Dayanır et al. compared three manual therapy techniques for chronic NSLBP; it involved myofascial trigger points (MTrPs) [32]. The signs of MTrPs were the existence of a taut band that was palpable in the muscle, a hyperirritable spot in the muscle band, a reaction of local twitch elicited on the palpation of the muscle, and the replication of pain upon palpation. Manual therapy techniques such as MTrP decrease pain severity, pressure pain threshold (PPT), and disability due to pain, depression, and anxiety and improve active range of motion (AROM) in patients with chronic NSLBP [32].

Many times, LBP is nonspecific. NSLBP can be managed by taking measures to avoid heavy lifting, correcting sitting posture, and doing exercises [2]. Money reviewed trigger points and myofascial pain syndrome, and their pathophysiology mentioned that MTrPs are tender spots in the muscle [33]. They are indications of myofascial pain, which involves stiffness of the muscle, pain, and tenderness that radiates to other locations, which are also described as referred pain. MTrPs are active or latent. Active trigger points inflict muscle pain most of the time, whereas MTrP, which are painful only on pressure, are latent. Trigger points are linked to muscle dysfunction, weakening, and reduced range of motion. One theory holds that trigger points are caused by muscle injury, overuse, and spasm. Another hypothesis is that trigger points are directly due to nerve pain from the spine. Yet, another theory is that maintaining incorrect posture for an extended period induces trigger points to occur [33].

Researchers could expand the study in the future by using INIT in a different group of muscles. A longer duration of treatment can be considered to achieve better results. This study had a smaller sample size of 80 and did not include an equal number of male and female samples. The therapist did not maintain follow-up in this study, which was a significant limitation.

## Conclusions

Living with LBP is something to be concerned about as it leads to functional disability and reduced range of motion. The most typical causes of LBP are MTrP, muscle tightness, and overuse injury. In this study, we compared the effect of INIT and Mulligan lumbar SNAG. The INIT group showed a reduction in pain and improvement in range of motion leading to decreased functional disability. Mulligan lumbar SNAG also proved to reduce pain and functional disability. When compared, it was seen that INIT helps significantly in reducing pain and functional disability. Thus, INIT was found more effective than MWM in managing NSLBP.
